# Exogenous mRNA delivery and bioavailability in gene transfer mediated by piggyBac transposition

**DOI:** 10.1186/1472-6750-13-75

**Published:** 2013-09-26

**Authors:** Solenne Bire, David Gosset, Gwenhael Jégot, Patrick Midoux, Chantal Pichon, Florence Rouleux-Bonnin

**Affiliations:** 1 LBTM, Institute of Biotechnology, UNIL-EPFL, Station 6, Lausanne 1015, Switzerland; 2 CBM-UPR CNRS, 4301 - Rue Charles Sadron Cedex 2, Orléans 45071, France; 3 Equipe BIOS, Unité PRC, INRA, Nouzilly 37380, France; 4 LNOX, GICC UMR CNRS 7292, UFR de Médecine, Bâtiment Dutrochet, 10 Boulevard Tonnellé, Tours 37000, France

**Keywords:** mRNA trafficking, Gene delivery, PiggyBac, Transposition, Bioavailability

## Abstract

**Background:**

Up to now, the different uptake pathways and the subsequent intracellular trafficking of plasmid DNA have been largely explored. By contrast, the mode of internalization and the intracellular routing of an exogenous mRNA in transfected cells are poorly investigated and remain to be elucidated. The bioavailability of internalized mRNA depends on its intracellular routing and its potential accumulation in dynamic sorting sites for storage: stress granules and processing bodies. This question is of particular significance when a secure transposon-based system able to integrate a therapeutic transgene into the genome is used. Transposon vectors usually require two components: a plasmid DNA, carrying the gene of interest, and a source of transposase allowing the integration of the transgene. The principal drawback is the lasting presence of the transposase, which could remobilize the transgene once it has been inserted. Our study focused on the pharmacokinetics of the transposition process mediated by the piggyBac transposase mRNA transfection. Exogenous mRNA internalization and trafficking were investigated towards a better apprehension and fine control of the piggyBac transposase bioavailability.

**Results:**

The mRNA prototype designed in this study provides a very narrow expression window of transposase, which allows high efficiency transposition with no cytotoxicity. Our data reveal that exogenous transposase mRNA enters cells by clathrin and caveolae-mediated endocytosis, before finishing in late endosomes 3 h after transfection. At this point, the mRNA is dissociated from its carrier and localized in stress granules, but not in cytoplasmic processing bodies. Some weaker signals have been observed in stress granules at 18 h and 48 h without causing prolonged production of the transposase. So, we designed an mRNA that is efficiently translated with a peak of transposase production 18 h post-transfection without additional release of the molecule. This confines the integration of the transgene in a very small time window.

**Conclusion:**

Our results shed light on processes of exogenous mRNA trafficking, which are crucial to estimate the mRNA bioavailability, and increase the biosafety of transgene integration mediated by transposition. This approach provides a new way for limiting the transgene copy in the genome and their remobilization by mRNA engineering and trafficking.

## Background

The secure insertion of a therapeutic gene into a defective cell is a major challenge for the gene-based treatment of many diseases. Transposon-based systems are particularly promising for use in integrating a gene of interest into the genome, since they are considered to be less immunogenic and to have a much larger cargo capacity than viral vectors, while maintaining highly efficient transgene integration. To date, few transposons have demonstrated their functionality in mammalian cells and their usefulness for reshaping the genome [1]. One that has, the piggyBac transposon, which is derived from the cabbage looper moth Trichoplusia ni, is mobile in many different species, including human cells [2]. This transposon has a higher cargo capacity [3] and is less susceptible to transposase overproduction inhibition of transposition [4] than Sleeping Beauty, another transposon widely used in mammalian cells.

Transposon vectors require two components: a plasmid DNA (pDNA) carrying the gene of interest, and a source of transposase. In the context of gene therapy, a secure transposon system able to integrate stably at least one copy of a therapeutic transgene per nuclear genome appears to be necessary. Optimizing pDNA architecture to ensure site directed integration of the transgene and long-term expression through the use of insulator has been described [5,6], but less has been characterized about the source of transposase. Usually this source is a pDNA carrying the transposase cDNA under the control of a strong promoter. The principal drawback encountered using this strategy is the lasting presence of the transposase due to persistence of the episomal pDNA and/or the possible non-specific integration of the transposase gene in the genome. This could remobilize the transgene once it has been inserted, and thus lead to genotoxicity. In an attempt to improve the biosafety of gene integration, we chose to deliver the source of transposase as a messenger RNA (mRNA), instead of the commonly used pDNA. Besides the lability of mRNA, the fact that it is not integrated into the genome eliminates the risk of long-lasting side effects. mRNA is attracting interest when only high levels and/or short-term expression are required to achieve the desired effect. Consequently, mRNA is of particular relevance for engineering secure transposon systems with limited transposase expression. In this context, transgenesis protocols based on Sleeping Beauty or piggyBac transposition providing the transposase in the form of mRNA have been validated in many eukaryotic species, including mouse and rat models [7-9], and in cultured human cells [10].

Despite the increasing use of mRNA as a promising alternative to pDNA for several therapeutic purposes, few transfection reagents have been developed for large RNA, such as mRNA. Cationic lipids are the ones most often used [11], since other reagents, including cationic peptides [12] or cationic polymers [13], have demonstrated poor efficiency in delivering mRNA, probably due to differences in the uptake and intracellular trafficking of mRNA formulations. So far, the various uptake pathways and subsequent intracellular trafficking of pDNA have been largely explored [14-16], whereas there have been very few investigations of the delineation of the intracellular fate of exogenous mRNA, which remains to be elucidated.

Our study focused on the pharmacokinetics of the transposition process mediated by the piggyBac transposase mRNA transfection of HeLa cells. We were interested in assaying different commercial chemical reagents for mRNA delivery. Then, in order to better apprehend the bioavailability of the transfected transposase mRNA, both spatially and temporally, we sought to shed light on exogenous mRNA uptake and its subsequent intracellular trafficking via endosomes and potential storage in Processing Bodies (P bodies) or Stress Granules (SG), which are cytoplasmic foci involved in natural, endogenous mRNA regulation. These aggregates can interact together and they constitute kind of dynamic sorting sites for storage and/or turnover of endogenous mRNA [17], where intracellular mRNA is routed to sites of translation reinitiation, degradation or storage [18].

Taken together, our results unveiling part of the process of mRNA incorporation, release and intracellular trafficking during transfection, would make it possible to better apprehend the spatio-temporal bioavailability of mRNA and improve the reliability of our transposon vector.

## Results and discussion

### Efficiency of transfection reagents for mRNA delivery and characterization of mRNA complexes

**Figure 1:**
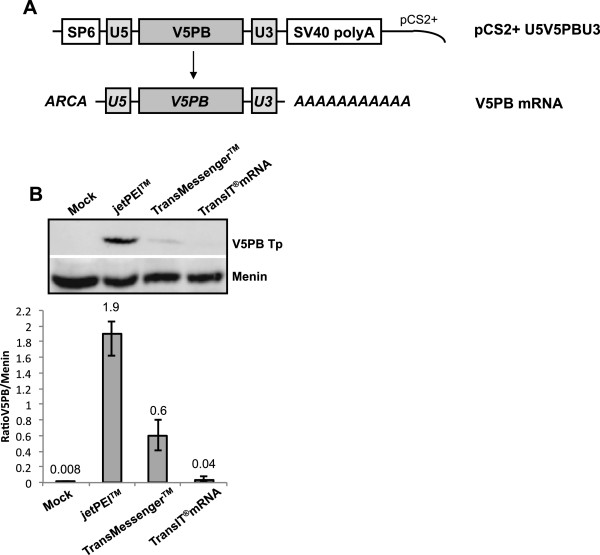
**Efficiency of transfection reagents for mRNA delivery. (A)** Diagram of a transposase vector plasmid and subsequent mRNA. The transposase gene sequence fused with the V5 epitope tag sequence (V5PB) and surrounded by the UTR5′ (U5) and UTR3′ (U3) sequences of the β-globin gene of the Xenopus laevis was cloned in the pCS2+ plasmid backbone to obtain the pCS2+ U5V5PBU3 construct. In vitro transcribed mRNA (V5PB mRNA) harbors a cap analogue (ARCA) and a polyA tail. **(B)** Efficiency of various different transfection reagents for mRNA delivery into HeLa cells. Cells were transfected with 200 ng of V5PB mRNA using jetPEI™ (N/P = 5), TransMessenger™ or TransIT®mRNA transfection reagents. Transfection efficacy was checked following protein expression by Western-Blot analysis 24 h post-transfection using an antiV5-Horseradish Peroxidase antibody to detect the tagged V5PB transposase (V5PB Tp). Protein quantification was normalized to the Menin endogenous protein (ratios are indicated). Data are the average of 4 experiments. Mock is a negative control without mRNA transfected.

Electroporation and micro-injection are the most widely used non-viral methods for delivering mRNA. Nevertheless, physical methods are both invasive and more cytotoxic than chemical methods, which also present the advantage of condensing and thus protecting nucleic acids from nucleases present in the cytoplasm; something which is of critical importance in the case of mRNA delivery. The mRNA used throughout this study was transcribed from the pCS2+ U5V5PBU3 plasmid in the presence of an anti-reverse cap analogue (ARCA) and then polyadenylated to enhance both the stability and the translatability, as described elsewhere [13,19] (Figure 1A). The ARCA allows capping with a modified cap (m^7^(3′-O-methyl)G(5′)ppp(5′)G), in which one of the 3′ OH groups is substituted with –OCH3. RNA polymerases can only initiate transcription with the remaining -OH group and thus synthesize RNA transcripts capped exclusively in the correct orientation.

Our first aim was therefore to identify an efficient commercially available transfection reagent for mRNA transfection. For this purpose, we used different formulations of transfection reagents, whether specially developed for mRNA transfection or not. Effective transfection was confirmed by quantifying the protein produced by Western-Blot analysis (V5PB Tp, Figure 1B) one day post-transfection. All carriers led to the expression of mRNA, but with clearly differing efficiencies. jetPEI™ was found to be the most effective reagent for delivering mRNA in HeLa cells, even though it had not been specifically designed for mRNA transfection according to the Manufacturer. mRNA transfection using TransMessenger™ and TransIT®mRNA, developed for mRNA delivery, produced only 30% and 2% respectively, of the transposase produced with jetPEI™_,_ which corresponds to 3-fold and 50-fold reductions of transfection efficiency in our conditions. Consequently, PEI was used in the following experiments to transfect mRNA in HeLa cells.

**Figure 2:**
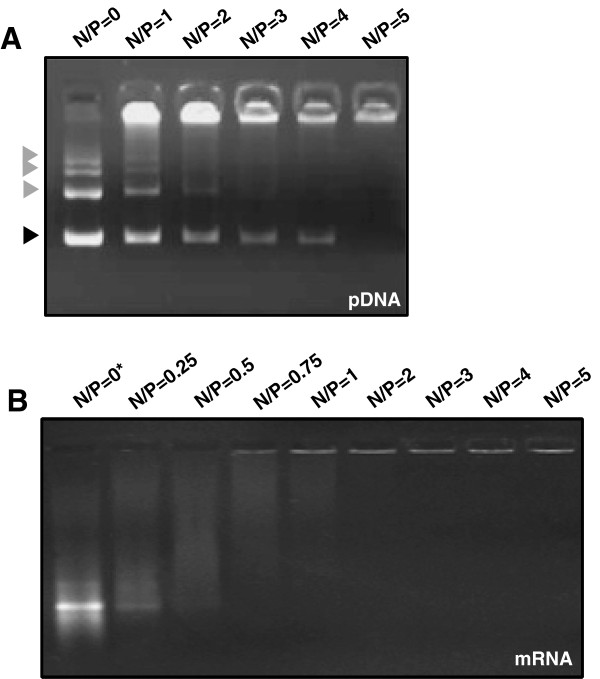
**Gel retardation assays. Electrophoretic migration of V5PB pDNA. (A)** or mRNA **(B)** complexed with PEI at varying N/P ratios ranging from 0 to 5. Complexes were prepared by mixing 1 μg of V5PB mRNA or pDNA with different amount of PEI according to the desired ratio. Black arrowhead indicates supercoiled pDNA whereas grey arrowheads indicate different states of condensation of nicked pDNA. Experiments have been done three times.

We then characterized mRNA complexes in vitro in order to better apprehend the behavior of such polyplexes in vivo. First, the condensation of the mRNA transposase (named V5PB) by PEI at various different N/P ratios was analyzed by gel retardation assay and compared to pDNA/PEI condensation (Figure 2). The N/P ratio refers to the number of nitrogen residues of PEI per DNA phosphate, it is a measure of the ionic balance of the complexes. It is well established that during electrophoresis, complexes, which are less negatively charged and heavier than free pDNA or mRNA, are retained in the wells whereas uncomplexed nucleic acids can migrate into the gel [20]. As shown in Figure 2A, the intensity of bands corresponding to free, supercoiled pDNA migrating in the gel decreased as the amount of PEI increased. At an N/P ratio of 5, all the pDNA was complexed and retained in the wells. Similar experiments were performed with V5PB mRNA (Figure 2B). Our results showed that an N/P ratio as small as 0.75 was sufficient to totally condense the mRNA, as no free mRNA migrated into the gel, attesting that PEI has greater affinity for mRNA than for pDNA, as described previously [12]. Even though an N/P ratio of 0.75 was sufficient to complex all the mRNA, the best transfection efficiency in cultured cells was obtained using an N/P ratio of 5, suggesting that free PEI is involved in the uptake and/or cytosolic delivery of polyplexes (Additional file 1). Finally, the size of V5PB mRNA/PEI polyplexes formed at an N/P ratio of 5 was determined by dynamic light scattering. The size distribution ranged from 140 to 300 nm, with a mean size of 190 nm (Additional file 2). This was consistent with results reported for pDNA polyplexes and polyplexes made with other mRNA [21].

Our data show that linear PEI polyplexes used at an N/P ratio of 5 are the most efficient formulation for promoting mRNA transfection and expression in HeLa cells. As we wanted to transfect plasmids and messenger RNA simultaneously, an N/P ratio of 5 was used to transfect pDNA and mRNA for the subsequent experiments.

### Accessibility and functionality of the complexed mRNA

**Figure 3:**
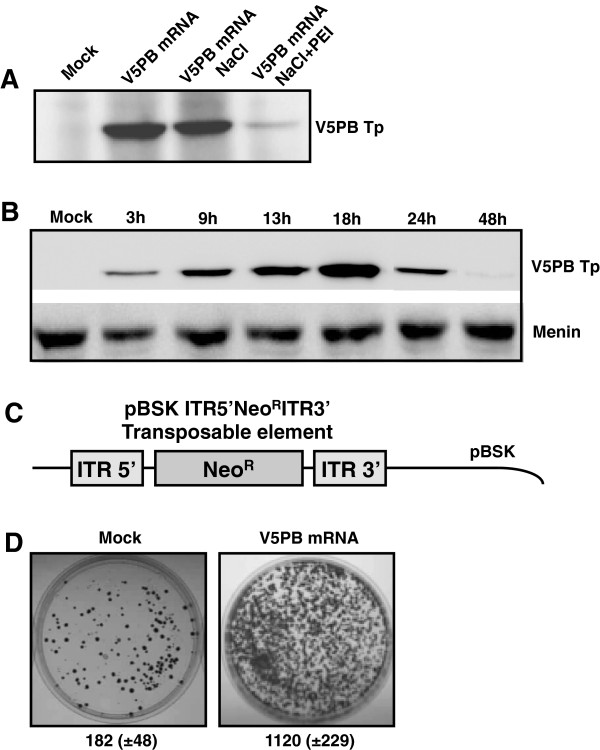
**mRNA/PEI availability for the in vitro and in vivo translation and functionality of V5PB transposase. (A)** In vitro translation of V5PB mRNA uncomplexed or complexed with PEI (N/P = 5). In vitro translation of mRNA alone (V5PB), in the presence of 150 mM NaCl (V5PB-NaCl) or complexed with PEI (V5PB-NaCl + PEI). Proteins V5PB Tp and Menin were separated by SDS-PAGE before chemiluminescent detection using an antiV5-Horseradish Peroxidase antibody. Mock is a negative control without mRNA. Experiments have been done in triplicate. **(B)** Time course of cellular production of V5PB transposase in HeLa cells (V5PB Tp). Transposase expression was assayed at the indicated time points, and monitored by Western-Blot experiments as described in **(A)**. Mock: negative control (untransfected cells). Experiments have been done in triplicate. **(C)** pBluescript plasmid (pBSK ITR5′Néo^R^ITR3′) harboring the transposable element used in this study. This vector was composed of the expression cassette encoding the neomycin phosphotransferase protein (Neo^R^) flanked by the inverted terminal repeats (ITR5′ and ITR3′), the recognition sites of the piggyBac transposase necessary for the transposition step. **(D)** Transposition assays in HeLa cells. Cells were transfected with the mRNA encoding the V5PB transposase and pBSK ITR5′Néo^R^ITR3′. Transposition events involve the integration of the Neo^R^ gene, and thus the emergence of a resistance phenotype to G418. Positive cells were selected under antibiotic pressure for 15 days, and colonies were then stained and counted. Mock is a negative control (without V5PB mRNA) corresponding to recombination events. The figures indicate the number of colonies counted (mean value ± standard deviation of three independent experiments made in triplicate).

The accessibility of mRNA for translation when formulated with PEI was assessed both in vitro and in cultured cells. In vitro translation was performed using a cell-free translation system based on rabbit reticulocyte lysate coupled with Western-Blot detection (Figure 3A). The efficiency of translation of free mRNA was compared to that of mRNA/PEI complexes. A control consisting of mRNA mixed with 150 mM NaCl excluded any potential salt effect during the in vitro translation step, as translation of mRNA mixed with 150 mM NaCl was equivalent to that with free mRNA. In contrast, the level of translation obtained with mRNA/PEI particles, was 9.5-fold lower than that obtained with free mRNA. These results suggest that condensation to PEI partially impedes, but does not completely inhibit, translation. Partial translation due to remaining free mRNA is excluded since at the N/P ratio of 5 used for this experiment, 100% of the mRNA was found to be complexed. The time course of translation into HeLa cells transfected with mRNA/PEI polyplexes was studied at different times (Figure 3B). Transposase production was detected as early as 3 h after transfection, peaked at 18 h and then declined. After 48 h, only traces were detected. This pattern may be explained by the progressive degradation or storage of the V5PB mRNA and/or protein in cytoplasmic foci. Indeed, quantitative RT-PCR studies indicated a half-life of the PB transposase mRNA of about 3 h, and western-blot analysis after incubating the V5PB mRNA transfected cells with cycloheximide, a known inhibitor of protein synthesis, indicated a half-life of the PB transposase of 12 h (manuscript in preparation). Overall, these data have shown for the first time that the mRNA prototype designed and characterized in this study provides a very short expression window of the *piggyBac* transposase.

Finally, the functionality of the transposase produced from our mRNA was assayed in a transposition assay. Briefly, HeLa cells were transfected with V5PB transposase mRNA and a donor plasmid carrying the gene of neomycin resistance (pBSK ITR5′Neo^R^ITR3′, Figure 3C). If functional, the transposase will integrate the neomycin resistance gene in the nuclear genome of cells, which could be selected through antibiotic pressure. Transfection of the V5PB mRNA led to a 6-fold increase in the colony number compared to the mock control indicating that transposase exhibits transposition activity (Figure 3D, right panel, 1120 colonies). In the absence of transposase, 182 colonies were observed corresponding to recombination events between the donor plasmid and the genome (Figure 3D, left panel). This is consistent with results obtained by Wilber et al. with Sleeping Beauty mRNA [10]. Consequently, the transfection of piggyBac mRNA by PEI enabled in vitro and cellular translation of a functional transposase, permitting transposition events to occur with high efficiency.

The cytotoxicity of different mRNA/PEI and pDNA/PEI polyplexes was evaluated 24 h and 48 h post-transfection using a proliferation assay. The mRNA or pDNA of piggyBac and green fluorescent protein (GFP) was tested to exclude the possibility of any impact of the nucleic acid sequence. No significant change of cell proliferation was detected when cells were treated with the carrier alone, suggesting that this carrier is safe and did not affect cell viability under these conditions (Additional file 3). Similarly, pDNA or mRNA condensed with PEI did not alter cell proliferation. These findings suggested that mRNA or pDNA complexed to PEI did not induce severe cell damage in HeLa cells under our conditions.

### Uptake pathways and subsequent intracellular trafficking of mRNA polyplexes

**Figure 4:**
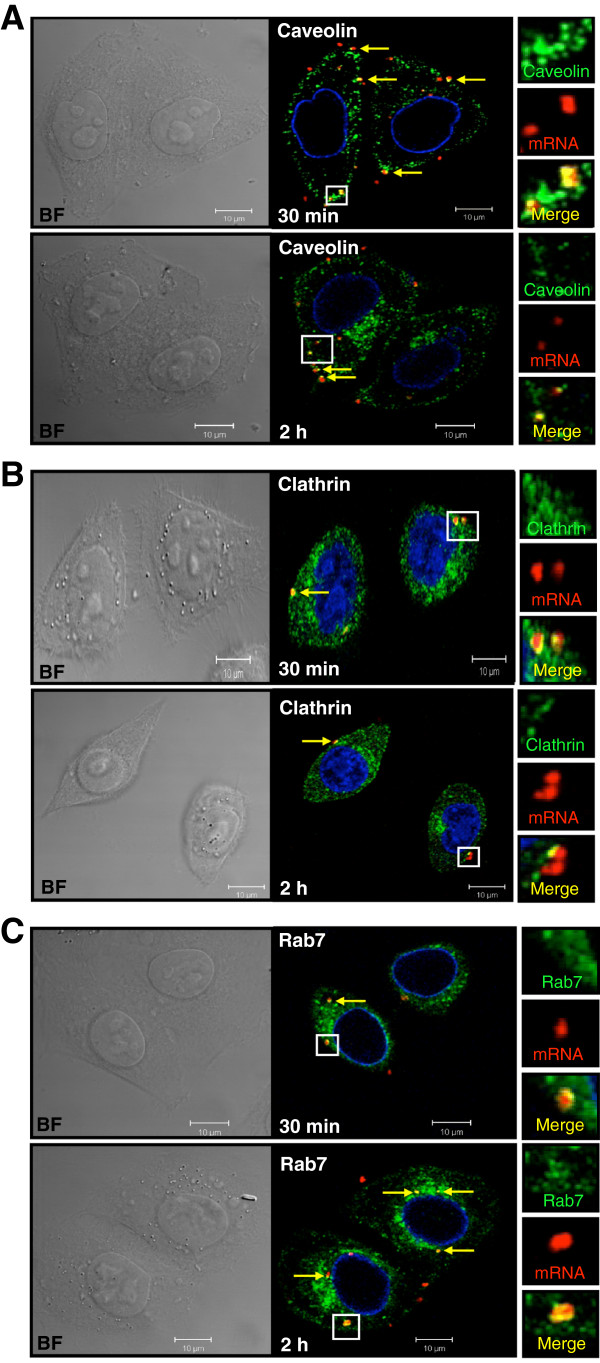
**Uptake pathways of mRNA polyplexes by HeLa Cells. (A)** Red fluorescent labeled V5PB mRNA compacted to PEI were administered to HeLa cells. Clathrin detection was performed by immunofluorescence using an anti-clathrin primary antibody revealed with an Alexa-488 conjugated secondary antibody (green). Experiments have been done three times. **(B and C)** Red fluorescent-labeled V5PB mRNA was used to transfect HeLa cells constitutively expressing green fluorescent caveolin-1 **(B)** or green fluorescent Rab7 proteins **(C)**. Experiments have been done three times. For all experiments, the route of the labeled polyplexes was observed at 30 min and 2 h post-transfection using confocal microscopy. Yellow dots correspond to colocalization of red and green fluorescent spots. Insets shows enlargement of boxed areas with colors separated. BF: Bright Field. Nuclei were stained in blue by lamin immunodetection (Caveolin and Rab7 experiments) or DRAQ5® (Clathrin experiment). The images shown are representative Z-sections from three separated experiments.

As the intracellular fate is linked to the entry mechanism, we studied the internalization routes of the mRNA polyplexes, which had never previously been investigated. We used confocal microscopy to reveal and quantify potential colocalizations between a red fluorescent-labeled V5PB mRNA and different markers of the endocytic pathways: caveolin-1 (caveolae), clathrin (clathrin-coated vesicles) and Rab7 (late endosomes) labeled with green fluorescence (Figure 4 and Table 1). Thirty minutes post-transfection, mRNA was colocalized with caveolin-1, indicating the involvement of the caveolae pathway in the uptake of mRNA/PEI polyplexes by HeLa cells. By 2 h post-treatment, slightly fewer complexes were visible in this compartment, the mean number of yellow dots dropped from 9.2 to 6.3 per cell (Table 1), suggesting that about 32% of mRNA polyplexes had started to escape from the caveolae vesicles into other compartments (Figure 4A). Similarly, mRNA polyplexes were found in clathrin vesicles, but fewer yellow dots were visible at both 30 min and 2 h (mean number of 2.6 and 2.3 yellow dots per cell, respectively), suggesting that the clathrin-mediated endocytosis is not the major route or is a slower route than the caveolae pathway for escaping from the early vesicles (Figure 4B). In both these compartments, the mean number of red dots is constant; they correspond to mRNA escaped from the endocytosis vesicles. mRNA was found in late endosomes as soon as 30 min post-transfection and yellow dots appeared bigger and six time more abundant after 2 h, in agreement with the endocytosis pathway (Figure 4C). Similarly, the mean number of red dots per cell is increasing over time, suggesting that mRNA is progressively released from late-endosomes. These observations demonstrate that the uptake of mRNA polyplexes of 200 nm in size at least involves two major endosomal pathways including both the caveolae and clathrin-coated pits in HeLa cells. This is consistent with the particle size supported by these endocytosis routes and the results obtained with pDNA complexed with PEI in this cell type [22,23]. Recently, Lorenz et al. have concluded that a huge amount of naked mRNA (20 μg) is taken up by a caveolae-dependent pathway, and to a minor degree by macropinocytosis, excluding any role of clathrin-coated pits [24]. Moreover, Diken et al. showed a dominant role of macropinocytosis in the uptake of naked RNA by immature dendritic cells in vitro and in vivo [25]. In our study, no fluorescent mRNA was observed inside the cells in the absence of PEI when a small amount of mRNA was used (0.5 μg), which is hardly internalized, as expected. No red fluorescent mRNA was observed in nuclei. Once internalized, some of the mRNA was directed to late endosomes, as has been reported for pDNA polyplexes [26]. At first glance, the mechanisms involved in mRNA internalization do not seem to differ from those involved in pDNA internalization in the early stages of transfection.

**Table 1:** Distribution of mRNA polyplexes in three endocytosis compartments

		**Mean number of red dots**	**Mean number of yellow dots**
**Caveolae (Caveolin-1)**	30m	9.3 (±3.5)	9.2 (±2.5)
	2h	8.5 (±5.3)	6.3 (±3.6)
**Clathrin-coated vesicle (Clathrin)**	30min	4.4 (±1.7)	2.6 (±1.5)
	2h	3.3 (±2.1)	2.3 (±1.5)
**Late endosome (Rab-7)**	30min	4.7 (±2)	1.1 (±0.4)
	2h	7.2 (±2.6)	6 (±3.5)

Red fluorescent-labeled mRNA polyplexes were observed in three green fluorescent endocytosis compartments 30 min and 3 h post-transfection. Yellow dots are obtained when there is colocalization. Each value represents the average number of detected dots for each color ± standard deviation per whole cell. Three separate experiments were performed and at least 20 cells were analysed using confocal microscopy and ImageJ software for each condition.

**Figure 5:**
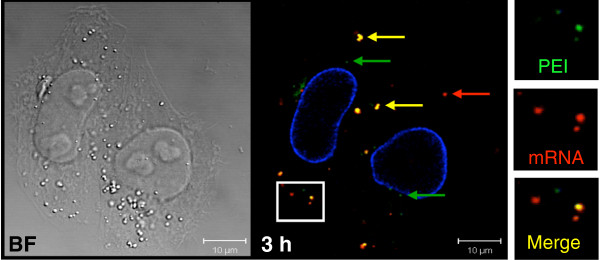
**Dissociation of mRNA/PEI polyplexes in HeLa cells.** Confocal microscopy images of HeLa cells incubated during 3 h with Alexa Fluor® 546-labeled mRNA (red) condensed with jetPEI™-FluoF (green). Yellow dots correspond to merge signal. Green spots match with jetPEI™-FluoF alone or complexed to unlabeled mRNA, and red spots correspond to dissociated mRNA from polyplexes. Experiments have been done three times. Insets show enlargements of the boxed areas with the colors separated. BF: Bright Field. Nuclei are stained in blue by lamin immunodetection. The images shown are representative Z-sections from three separated experiments. No red fluorescent mRNA was observed in nuclei.

PEI is thought to facilitate the release of the polyplexes into the cytoplasm thanks to a proton sponge effect, which involves disruption of the late endosome through its acidification, avoiding acidic lysosomes [27]. So, to keep track of mRNA/carrier interactions during navigation through the cytosol, red fluorescent mRNA was condensed with green fluorescent PEI and incubated with HeLa cells. In Figure 5, an analysis carried out 3 h post-transfection revealed the presence of: (i) cytoplasmic yellow dots corresponding to dual fluorescent polyplexes, indicating that mRNA and the carrier were still either compacted or localized together; (ii) green spots related to PEI either alone or complexed to unlabeled mRNA and (iii) red spots corresponding to mRNA released from PEI. These data show that some of the internalized mRNA was released from polyplexes 3 h post-transfection, which corresponds to the beginning of the transposase production (Figure 3B). It should be noted that direct translation of mRNA/PEI complexes cannot be ruled out, even though it may occur less efficiently than naked mRNA. The quantification of the dots for each color given in Table 2 indicates that 47% of the mRNA still co-localized with PEI 3 h post-transfection (4.8 yellow dots per cell versus 1.6 red dots per cell), suggesting that mRNA release (18%) probably started at this observed time. The mechanisms responsible for mRNA/PEI dissociation, and where exactly this phenomenon takes place (in the late endosome or cytosol) remain to be elucidated.

**Table 2:** Dissociation of mRNA/PEI polyplexes

	**Mean number of red dots**	**Mean number of green dots**	**Mean number of yellow dots**
**mRNA/PEI polyplexes**	1.9 (±1.7) 18%	3.6 (±1.8) 35%	4.8 (±3.1) 47%

Dissociation of red fluorescent-labeled mRNA from green fluorescent PEI polyplexes 3 h post-transfection. Yellow dots are obtained when there is colocalization. Each value represents the average number of detected dots for each color ± standard deviation per whole cell. Three separate experiments were performed and at least 20 cells were analysed using confocal microscopy and ImageJ software for each condition.

**Figure 6:**
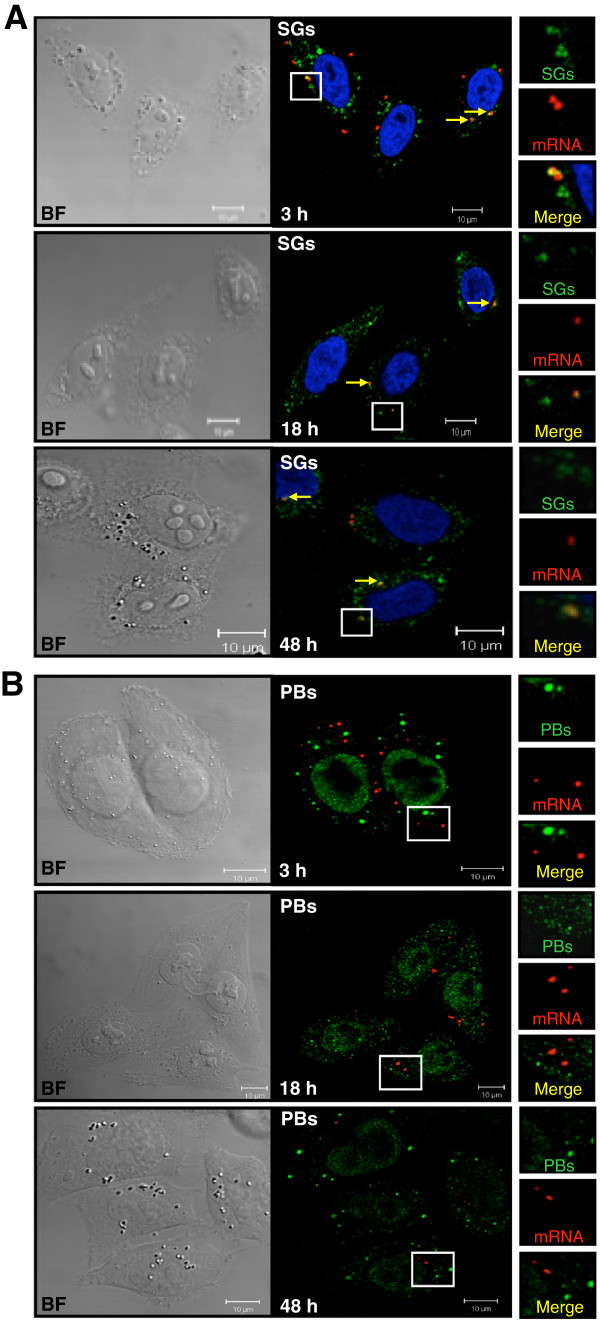
**mRNA localization in subcellular foci.** HeLa cells were observed using confocal microscopy 3 h, 18 h and 48 h post-transfection with 500 ng of red fluorescent-labeled V5PB mRNA polyplexes. Experiments have been done three times. GE-1/Hedls (P bodies marker) **(A)** and the eukaryotic initiation factor-3 eIF3η (Stress Granules marker, SGs) **(B)** were immunodetected using specific green fluorescent secondary antibodies. Insets show enlargements of the boxed areas with separated colors. Arrows indicate representative foci. The primary antibody directed against the nuclear p70 S6 kinase protein reveals P bodies foci due to cross-reactivity with GE-1/Hedls, and exhibits nuclear staining due to p70 S6 kinase protein detection. Nuclei were stained in green **(A)** or blue by DRAQ5® **(B)**. BF: Bright Field. The images shown are representative Z-section from three separated experiments. No re5d fluorescent mRNA was observed in nuclei.

Finally, we determined where the transfected mRNA ends up once it has escaped from late endosomes, whether it is still complexed with PEI or not. As the above data suggest that some of our exogenous mRNA could be available for translation in a condensed or free state, we investigated whether the other fraction could be stored in subcellular compartments, such as stress granules (SG), which are sorting sites for endogenous intracellular mRNA generated during cellular stress, such as transfection [28], and processing bodies (P bodies), which are involved in mRNA degradation [17,29]. Note that not all mRNA is intended to be degraded within these foci, as translationally repressed mRNA can also exit SG and P bodies to re-engage in translation [30]. Potential colocalizations of mRNA with SG and P bodies were assessed 3 h, 18 h and 48 h post-transfection using red fluorescent V5PB mRNA, and green-labeled SG and P bodies markers (Figure 6).

As shown in Figure 6A, SG were present throughout the cytoplasm 3 h post-transfection. A mean number of 3.1 merge spots per cell (Table 3) indicated that a part of the exogenous mRNA was found in SG at this time. The major part of mRNA did not colocalize with SG (18.4 red dots per cell in average) when some mRNA was found to be dissociated from PEI and translated. Fewer merge dots were observed at 18 h and 48 h post-transfection (1.5 yellow dots per cell in average), suggesting lower but prolonged storage in these foci (Figure 6A and Table 3). It is not known whether the mRNA directed to SG is still functional. Nevertheless, it was found that transposase mRNA sequestration at 3 h is not efficient enough to hinder the transposase production and transposition (Figure 3). The mean number of red dots per cell decreased over time, suggesting a degradation of the nucleic acids, which is consistent with the half-life of the V5PB mRNA and the kinetic of transposase expression (Table 3 and Figure 3B). We showed here for the first time that transfected mRNA could be directed to these specific granules, where it could be stored for subsequent translation. SG could be involved in the availability of exogenous mRNA by influencing its cytoplasmic retention and/or its subsequent distribution. This could explain why, in spite of a short half-life, traces of V5PB mRNA could be detected for 2 days, probably due to its sustained release over time. However, the decrease in protein expression from 18 h post-transfection despite the storage of transposase mRNA in SG indicates that this mechanism is not sufficient for prolonged protein production. This argues in favor of transient transposase expression and thus, stable transgene integration.

**Table 3:** Distribution of exogenous mRNA in sub cellular foci

		**Mean number of red dots**	**Mean number of yellow dots**
**Stress granules**	3h	18.4 (±5.9)	3.1 (±1.8)
	18h	6 (±2.4)	1.5 (±0.8)
	48h	2.6 (±0.9)	1.5 (±0.5)
**P bodies**	3h	10.8 (±4.6)	0
	18h	8 (±2.8)	0
	48h	1.7 (±0.7)	0

Distribution of red fluorescent-labeled mRNA in green subcellular foci 3 h, 18 h and 48 h post-transfection. Yellow dots are obtained when there is collocation. Each value represents the average number of detected dots for each color ± standard deviation per whole cell. Three separate experiments were performed and at least 20 cells were analysed using confocal microscopy and ImageJ software for each condition.

No colocalization with P bodies was detected 3 h, 18 h and 48 h post-transfection despite observation of spots corresponding to P bodies and to mRNA alone in the cytoplasm. This indicates that no exogenous mRNA coalesced in these foci at these times (Figure 6B). As for SG observations, the mean number of red dots was decreasing from 10.8 to 1.7 spots per cell over time, suggesting mRNA degradation (Table 3). As theV5PB mRNA was engineered for enhanced stability, the absence of P bodies addressing elements such as AU-rich elements, 5′ stem loops, premature translation termination codons, introns or short poly(A) tails, could explain why it was not directed to these foci [18]. Another explanation could also be the residence time in such granules. Indeed, it has been demonstrated that the transit time of mRNA in SG does not exceed 60 s [31]. This rapid exchange may also occur with P bodies, making it difficult to catch colocalization, especially if such an event is rare. Similarly, the number of colocalization events between mRNA and SG marker observed could have been underestimated. In HeLa cells, the chemical half-life of V5PB transposase mRNA was found to be 3 h, which is quite short considering the fact that stabilizing sequences (cap, polyA tail and β-globin untranslated regions (UTR)) had been added. As our mRNA does not coalesce in P bodies, this could suggest that the mechanisms responsible for exogenous mRNA degradation probably do not depend on the decapping pathway.

## Conclusions

**Figure 7:**
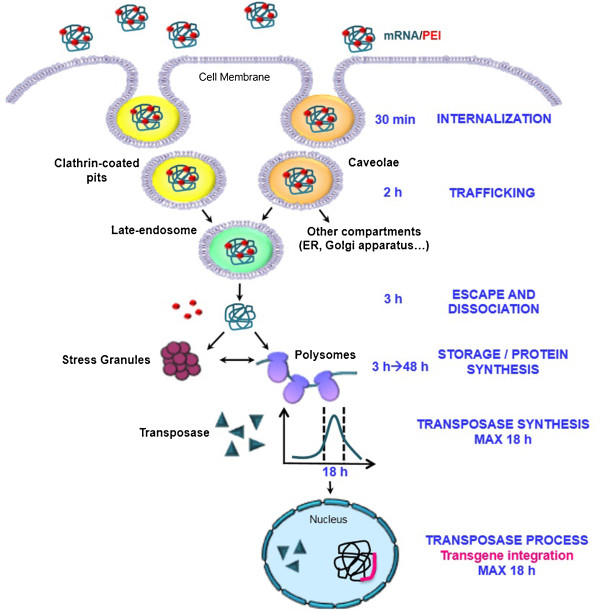
**Model of the trafficking of exogenous mRNA.** V5PB mRNA polyplexes could be both internalized by clathrin- and caveolin-mediated endocytosis 30 min after the cell treatment. Polyplexes in clathrin-coated vesicles are then directed to late endosomes 2 h post-transfection. One fraction of the mRNA polyplexes engulfed by caveolae is also targeted to late endosomes, whereas another fraction, trapped in caveosomes, is assumed to end up in other cytoplasmic compartments, such as the endoplasmic reticulum (ER) or the Golgi apparatus. mRNA polyplexes escape from the late endosome before its fusion with lysosomal vesicles thanks to the PEI proton sponge effect 3 h after cell transfection. PEI and mRNA dissociate from each other. Some of the V5PB mRNA can reach the translational machinery (polysomes) for protein synthesis, as demonstrated by transposase expression, whereas other fractions may be directed to RNA stress granules for transient storage.

Taking together, results reported in this study shed new light on the bioavailability of the piggyBac transposase, and the security of the transposition process by studying exogenous mRNA transfection and its underlying intracellular trafficking and fate. This offers several starting points of special relevance for any study requiring mRNA delivery. A schematic model based on our results and on observations in the literature is proposed in Figure 7.

In the transposition process, in vitro transcribed mRNA delivery and accessibility are satisfactory, as only short-term expression is required. Moreover, mRNA lability property due to rapid degradation by ubiquitous RNases and lack of integration into the genome eliminates the risk of long-lasting side effects and makes it clinically safer than pDNA for transposase expression. Nevertheless, for other applications, such as vaccination and the generation of induced pluripotent stem cells, greater mRNA availability would be required. mRNA could be engineered as needed to affect its expression and stability by using optimized cap analogue and poly(A) tails of variable length or by adding 3′UTR of various origins [13,19,32]. Finally, the sequence of the mRNA could be modulated to limit addressing to RNA granules, and produce mRNA "resistant" to SG or P bodies [18].

A lot still needs to be done to improve mRNA uptake and expression, but the increased knowledge that has been acquired these recent decades about pDNA delivery could be fully exploited during the first steps of mRNA transfection. To conclude, exact knowledge of spatial and temporal control of mRNA bioavailability is crucial to achieve improved biosafety of transposon vectors, which is a pre-requisite for the purpose of secure ex vivo gene and cell therapies, but it will also provide the basis for improving transfection efficiency and for designing novel devices for the cytosolic delivery of mRNA, and be beneficial for the development of many other emerging mRNA-based therapies.

## Methods

### Plasmid constructs

#### Helper plasmid

The construct designated V5PB pDNA (6.4 kbp) was made as follows: the 3′UTR fragment of the Xenopus laevis β-globin gene (designated U3, 317 bp) was removed from pBSSK/SB10 (kindly supplied by Dr.Z. Ivics, Max Delbrück Center, Germany), and inserted into pCS2+ already containing the 5′UTR (designated U5) (Invitrogen/Life Technologies, Paisley, UK). The piggyBac transposase fused with the V5 epitope tag (V5PB) (2067 bp) [GenBank accession number: EF587698, [33] was synthesized by ATG:biosynthetic (Merzhausen, Germany), and cloned in the pCS2+ U5U3 backbone between the UTRs. A negative control plasmid designated GFP pDNA (5.1 kbp) was built by ligation of the green fluorescent protein coding sequence (800 bp) between the UTRs of the pCS2 + U5U3 construct.

#### Donor plasmid

pBSK- ITR5′-Neo-ITR3′ (5.2 kbp) was generated by introducing the piggyBac 5′ and 3′ Inverted Terminal Repeats (ITRs, 717 bp) into the pBluescript SK- plasmid (Agilent Technologies, Santa-Clara CA, USA). The neomycin phosphotransferase gene (1515 bp) under control of the SV40 promoter was then inserted between the ITRs.

### In vitro mRNA synthesis

V5PB pDNA or GFP pDNA templates for mRNA synthesis were linearized by NotI digestion and followed by proteinase K treatment and phenol/chloroform extraction. In vitro transcription of capped mRNA was performed onto the linear templates according to the Manufacturer′s instructions using the MEGA Script SP6 kit (Ambion/Applied Biosystems, Austin TX, USA) combined with the ARCA (Anti-Reverse Cap Analog kit, Ambion). The resulting mRNAs were therefore polyadenylated using a Poly(A) Tailing kit (Ambion), and finally suspended in nuclease-free water after lithium chloride precipitation. The quality was checked by 0.8% agarose gel electrophoresis, and the mRNA concentration was determined by measuring the absorbance at 260 nm before storage at -80°C. piggyBac and GFP mRNAs are named V5PB mRNA (2.5 kb) and GFP mRNA (1.3 kb) respectively. Fluorescent labeled V5PB mRNA was produced using 2 μL of ChromaTide® Alexa Fluor® 546-14-UTP (red fluorescence, λEx: 550 nm, λEm: 570 nm, Invitrogen) during an in vitro transcription reaction.

### Cell lines and culture

HeLa cells (ATCC CCL-2) were cultured in 75 cm^2^ flasks (PAA Laboratories, Pasching, Austria) in DMEM supplemented with 10% fetal bovine serum (PAA Laboratories), 275 units of penicillin, and 275 units of streptomycin at 37°C in a humidified 5% CO_2_-containing atmosphere. HeLa-caveolin-1-eGFP and Hela-Rab7-eGFP, which are clones that constitutively express caveolin-1 or Rab7 fused with eGFP, were cultured in the same medium, but in the presence of 100 μg/mL of gentamicin.

### Nucleic Acid (NA) transfection

Cells were plated at a density of 1.10^5^ cells per well in 24-well plates and grown for 24 h to 80% confluence. Transfections using TransMessenger™ (Qiagen, Hilden, Germany) or TransIT®mRNA (Mirus Bio, Madison WI, USA) were performed following the manufacturer’s instructions. Transfections using jetPEI™ or fluorescein-labeled jetPEI™-FluoF (Polyplus Transfection, Illkirch, France) were performed at an N/P ratio of 5 in Opti-MEM (Invitrogen) following the Manufacturer’s protocol. When mRNA and pDNA were co-transfected, complexes were formed separately with the transfection reagent to avoid mRNA degradation due to RNases potentially present in the pDNA preparation. Cells were then incubated at 37°C for 30 min to 48 h, depending on the experiment.

### Gel retardation assay of mRNA complexes

One microgram of transposase mRNA and the appropriate amount of the jetPEI™ were supplemented with 150 mM sodium chloride to 25 μL and vortexed. The diluted jetPEI™ was added to the mRNA solution at different N/P ratios and vortexed. After 30 min of incubation at room temperature, the complex solution (50 μL) was mixed with 5 μL loading buffer and applied to a 0.8% agarose gel containing ethidium bromide (0.1 μg/mL). Electrophoresis was performed with a current of 90 V for 1 h in autoclaved TAE as running buffer and mRNA localization was visualized by ultraviolet transillumination.

### Western-blot

Cells were transfected using 200 ng of V5PB mRNA and a total protein extraction was performed 24 h post-transfection with 500 μL of extraction buffer containing 0.5% SDS, 100 mM NaCl, 10 mM ß-Mercaptoethanol and 1X protease inhibitor. Cell lysate was sonicated twice for 10 s, heated for 5 min to 95°C, and centrifuged for 5 min at 10,000 rpm at room temperature. 15 μg of protein extract supernatant were loaded per lane in a 4-10% gradient SDS-PAGE gel. The gel was transferred to a Hybond-ECL™ membrane (GE Healthcare, Little Chalfont, UK), and the membrane was blocked with 5% non-fat dry milk dissolved in PBS-Tween20 0.05% for 1 h at room temperature. It was then incubated overnight with a mouse anti-V5 HRP antibody (Invitrogen) diluted 1/5000. Specific piggyBac transposase (known as V5PB Tp) bands were detected using an ECL™ Western-Blot Analysis System (GE Healthcare) and an LAS-4000 apparatus (Fujifilm, Tokyo, Japan). Protein quantification was performed with the MultiGauge 4.0 software, and normalized to an endogenous protein: Menin, detected using a home-made antibody.

### In vitro translation of mRNA transcripts

In vitro translation of in vitro transcribed mRNA was performed according to the Manufacturer’s instructions using a nuclease-treated Rabbit Reticulocyte Lysate System (Promega, Madison WI, USA). Briefly, 2 μg of transposase mRNA, either free or condensed by jetPEI™ in NaCl 150 mM, were added to the reaction mix and translation products were separated by SDS-PAGE before chemiluminescent detection of the V5 tagged transposase using an anti-V5 horseradish peroxidase antibody as described above.

### Transposition assay

Cells were co-transfected with 200 ng of V5PB mRNA and with equal amounts of donor plasmid to produce a 1:1 ratio. Two days after transfection, cells were transferred to 100 mm plates followed by G418 selection (800 μg/mL, PAA Laboratories) for 15 days. Colonies were fixed and stained with 70% ethanol-0.5% methylene blue for counting. Only colonies >0.5 mm in diameter were counted.

### Immunofluorescence and confocal laser scanning microscopy

HeLa cells were seeded at 6.10^4^ cells per well in 24-well plates containing glass cover slips. On the next day they were transfected with 500 ng of red fluorescent-labeled mRNA. Cells were fixed with 4% p-formaldehyde, and cover slips were mounted on slides using Vectashield mounting medium (Vector Laboratories, Burlingame CA, USA) for confocal microscopy observation.

mRNA/PEI co-localization. Cells were transfected with mRNA complexed with jetPEI™-FluoF, a green fluorescent-labeled jetPEI™ at an N/P ratio of 5. Cells were fixed at 30 min or 3 h post transfection and mounted.

mRNA endocytosis. HeLa-caveolin-1-eGFP and Hela-Rab7-eGFP were transfected with V5PB mRNA complexed with jetPEI™ (N/P of 5). 30 min or 2 h post-transfection, cover slips were fixed and mounted. For experiments involving the clathrin pathway, cells were fixed and permeabilized for 15 min with 0.5% Triton-X 100 in PBS. Mouse anti-Clathrin HC primary antibody (Santa Cruz Biotechnology, Santa Cruz CA, USA, sc-271252) was then added for 1 h at room temperature, followed by a 45 min incubation with Alexa Fluor® 488 goat anti-mouse IgG (H + L) secondary antibody (Invitrogen) before mounting. Primary and secondary antibodies were diluted in 1% BSA-PBS blocking solution.

mRNA and stress granules or processing bodies colocalizations. HeLa cells were transfected as described above. Three hours, 18 h or 48 h post-transfection, cells were treated with 5 mg/mL digitonin for 5 min, fixed and permeabilized. GE-1/hedls and eIF3η markers were chosen to detect processing bodies and stress granules, respectively [28]. The GE-1/hedls marker can be detected using an anti-p70 S6 kinase monoclonal antibody, which exhibits strong cross-reactivity towards GE-1/hedls proteins situated in cytoplasmic P bodies and p70 S6 kinase located in the nucleus [28]. Mouse anti-p70 S6 kinase primary antibody (Santa Cruz Biotechnology, sc-8418) for the detection of P bodies, or goat anti-eIF3η primary antibody (Santa Cruz Biotechnology, sc-16377) for the detection of stress granules, was added for 1 h at room temperature. Cells were then incubated with Alexa Fluor® 488 goat anti-mouse IgG (H + L) (Invitrogen) or donkey anti-goat IgG-FITC (Santa Cruz Biotechnology, sc-2024) secondary antibodies before mounting. Primary and secondary antibodies were diluted in 1% BSA-PBS blocking solution.

Nuclei were stained in blue using DRAQ5® (5 μM, 30 min, Cell Signaling Technology, Danvers MA, USA) for experiments with clathrin and stress granules. Nuclei were stained in blue using a Goat anti-Lamin A/C (N-18) sc-6215 primary antibody (Santa Cruz Biotechnology), coupled with donkey anti-goat IgG-PerCP-Cy5.5: sc-45102 (Santa Cruz Biotechnology) for experiments with caveolin-1, Rab7 and jetPEI™-FluoF. Nuclei were stained green due to cross-reactivity of the primary antibody with the nuclear p70 S6 kinase in the experiments with P bodies. All antibodies used showed successful specific isotype controls since treating cells with the secondary antibodies alone revealed no signal.

Slides were analyzed using an LSM 510 META scanning device coupled to an Axiovert 200 microscope (Carl Zeiss, Oberkochen, Germany). Each experiment was performed three times, and representative images were obtained from randomly-selected areas, all analyzed using Z-sectioning. Images were processed using the routine software of the LSM 510 and ImageJ.

## Abbreviations

ARCA: Anti reverse cap analog; BSA: Bovine serum albumin; BF: Bright field; ER: Endoplasmic reticulum; GFP: Green fluorescent protein; ITR: Inverted terminal repeat; mRNA: messenger RNA; MTT: 3-(4,5-Dimethylthiazol-2-yl)-2,5-diphenyltetrazolium bromide; P bodies: Processing bodies; PEI: Polyethyleneimine; PBS: Phosphate buffer saline; SG: Stress granules; pDNA: plasmid DNA; SDS-PAGE: Sodium dodecyl sulfate polyacrylamide gel electrophoresis; UTR: Untranslated region.

## Competing interests

The authors declare they have no competing interests.

## Authors’ contributions

SB performed all experiments. DG is expert in confocal microscopy. GJ initiated the work on mRNA. PM aided in the design of the study. SB, FRB and CP conceived the study and guided the design of experiments. SB, FRB, CP analyzed the data. SB and FRB wrote the paper. All authors read and approved the final manuscript.

## Supplementary Material

Additional file 1 **N/P ratio alternative.** Method: 1.10^5^ HeLa cells were transfected with 500 ng of GFP mRNA using PEI during 3 h. After transfection incubation, cells were washed three times with PBS before microscopy observation using Nikon Eclipse Ti and NIS-Elements software. Figure legend: Cells are transfected with 500 ng of GFP mRNA using PEI at N/P ratio of 1 (A); 3 (B); 4 (C) or 5 (D) and protein expression is analyzed by epifluorescence microscopy with the x10 optical. Higher transfection efficiency is obtained using a N/P ratio of 5. Click here for file

Additional file 2 **Determination of the size of the complexes.** Method: V5PB mRNA/PEI complexes were prepared in a final volume of 1.4 mL of 150 mM NaCl using 14 μg of transposase mRNA, and the appropriate amount of jetPEI™ (N/P ratio of 5). The average particle size of the polyplexes was determined twice by dynamic light scattering using the Zetasizer 3000 (Malvern Instruments, Malvern, UK) with the following specifications: automatic sampling time, 10 measurements per sample, medium viscosity 0.89 cP, refractive index medium 1.33, temperature 25°C. Figure legend: Determination of the size of PEI-V5PB mRNA conjugates at an N/P ratio of 5. (A) Particles size, intensity, volume and number were determined by dynamic light scattering after 30 min of condensation. (B) Peaks analyses by intensity, volume and number. Data show two peaks with an average size of approximately 190 nm and 2150 nm. The first peak correspond to mRNA complexed with PEI, the second peak correspond to mRNA alone (uncomplexed or released over time due to the long time needed for data acquisition). Click here for file

Additional file 3 **Cell proliferation assay.** Method: Cell proliferation was evaluated 24 h and 48 h post-transfection of 1.10^4^ cells by performing an MTT ((3-(4,5-Dimethylthiazol-2-yl)-2,5-diphenyltetrazolium bromide) assay according to manufacturer’s instructions (CellTiter96® Non-Radioactive Proliferation assay, Promega). Briefly, the culture medium was replaced by 100 μL of fresh medium supplemented with 15 μL Dye solution. After 4 h incubation at 37°C, 100 μL Solubilization/Stop solution were added followed by further 1 h incubation at 37°C. Sample absorbance and background absorbance were measured at 595 nm and at 650 nm with a spectrophotometer (Biophotometer Plus, Eppendorf, Hamburg, Germany). The cell viability was calculated by subtracting the absorption at 650 nm (background absorbance) from the absorption at 595 nm. The viability of non-transfected cells was set as 100% as a standard. Figure legend: Viability of HeLa cells after transfection with PEI/mRNA or pDNA. Cells were transfected using PEI (N/P = 5) alone or with 200 ng of V5PB or GFP mRNA or pDNA. One or two days post transfection, cell viability was assayed using the CellTiter96® Non-Radioactive Proliferation MTT assay. Values obtained for cells without treatment (mock) were set to 100%. Values represent the means ± SD (n = 4). Click here for file
