# Supplementary figures and images for: Exogenous mRNA delivery and bioavailability in gene transfer mediated by piggyBac transposition
Source: BMC Biotechnol. 2013 Sep 26;13:75. doi: 10.1186/1472-6750-13-75 (PMC3849706; doi:10.1186/1472-6750-13-75)

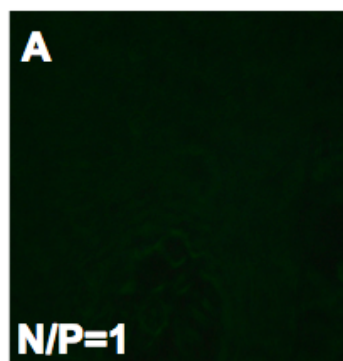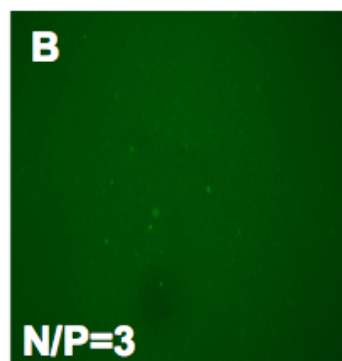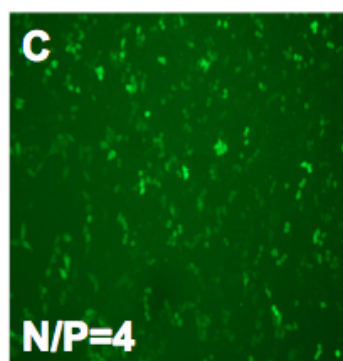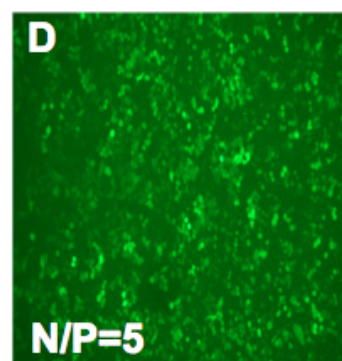

Supplement: Additional file 1 — N/P ratio alternative. Method: 1.105 HeLa cells were transfected with 500 ng of GFP mRNA using PEI during 3 h. After transfection incubation, cells were washed three times with PBS before microscopy observation using Nikon Eclipse Ti and NIS-Elements software. Figure legend: Cells are transfected with 500 ng of GFP mRNA using PEI at N/P ratio of 1 (A); 3 (B); 4 (C) or 5 (D) and protein expression is analyzed by epifluorescence microscopy with the x10 optical. Higher transfection efficiency is obtained using a N/P ratio of 5. [file 1472-6750-13-75-S1.pdf]

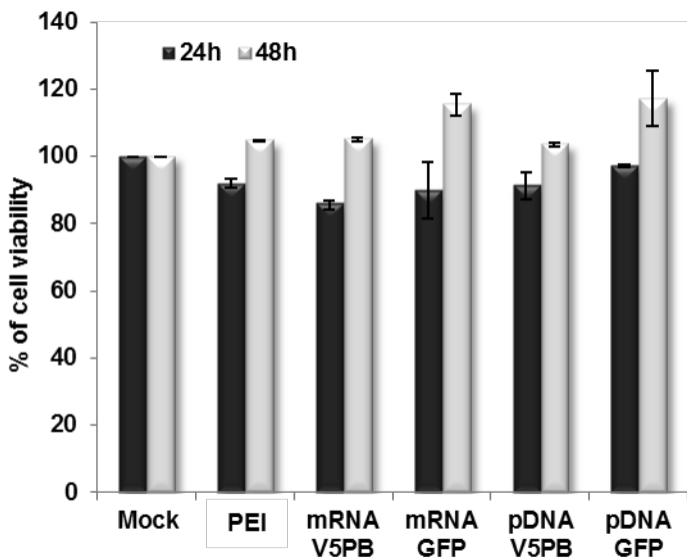

Supplement: Additional file 3 — Cell proliferation assay. Method: Cell proliferation was evaluated 24 h and 48 h post-transfection of 1.104 cells by performing an MTT ((3-(4,5-Dimethylthiazol-2-yl)-2,5-diphenyltetrazolium bromide) assay according to manufacturer’s instructions (CellTiter96® Non-Radioactive Proliferation assay, Promega). Briefly, the culture medium was replaced by 100 μL of fresh medium supplemented with 15 μL Dye solution. After 4 h incubation at 37°C, 100 μL Solubilization/Stop solution were added followed by further 1 h incubation at 37°C. Sample absorbance and background absorbance were measured at 595 nm and at 650 nm with a spectrophotometer (Biophotometer Plus, Eppendorf, Hamburg, Germany). The cell viability was calculated by subtracting the absorption at 650 nm (background absorbance) from the absorption at 595 nm. The viability of non-transfected cells was set as 100% as a standard. Figure legend: Viability of HeLa cells after transfection with PEI/mRNA or pDNA. Cells were transfected using PEI (N/P = 5) alone or with 200 ng of V5PB or GFP mRNA or pDNA. One or two days post transfection, cell viability was assayed using the CellTiter96® Non-Radioactive Proliferation MTT assay. Values obtained for cells without treatment (mock) were set to 100%. Values represent the means ± SD (n = 4). [file 1472-6750-13-75-S3.pdf]
