# Supplementary material for: Exogenous mRNA delivery and bioavailability in gene transfer mediated by piggyBac transposition
Source: BMC Biotechnol. 2013 Sep 26;13:75. doi: 10.1186/1472-6750-13-75 (PMC3849706; doi:10.1186/1472-6750-13-75)
Supplement: Additional file 2 — Determination of the size of the complexes. Method: V5PB mRNA/PEI complexes were prepared in a final volume of 1.4 mL of 150 mM NaCl using 14 μg of transposase mRNA, and the appropriate amount of jetPEI™ (N/P ratio of 5). The average particle size of the polyplexes was determined twice by dynamic light scattering using the Zetasizer 3000 (Malvern Instruments, Malvern, UK) with the following specifications: automatic sampling time, 10 measurements per sample, medium viscosity 0.89 cP, refractive index medium 1.33, temperature 25°C. Figure legend: Determination of the size of PEI-V5PB mRNA conjugates at an N/P ratio of 5. (A) Particles size, intensity, volume and number were determined by dynamic light scattering after 30 min of condensation. (B) Peaks analyses by intensity, volume and number. Data show two peaks with an average size of approximately 190 nm and 2150 nm. The first peak correspond to mRNA complexed with PEI, the second peak correspond to mRNA alone (uncomplexed or released over time due to the long time needed for data acquisition). [file 1472-6750-13-75-S2.pdf]

A

Size (nm)	Intensity	Volume	Number
0	0	0	0
140.5	7.8	8.9	8.9
176.9	12.2	10.1	10.1
222.8	8	7.5	7.5
280.5	1.7	2.9	2.9
353.1	0	0.4	0.4
444.6	0	0	0
559.7	0	0	0
704.7	0	0	0
887.3	0	0.8	0.8
1117.2	3.3	4.4	4.4
1406.6	10.8	10.6	10.6
1770.9	17.3	15.9	15.9
2229.7	18.1	16.7	16.7
2807.3	13.4	12.6	12.6
3534.5	5.6	6.2	6.2
4450.1	0	1.4	1.4
5602.9	0	0	0
7054.3	0	0	0
8881.7	0	0	0
11182.4	0	0	0
14079.2	0	0	0

B

Peak analysis by intensity			
Peak	Area	Mean	Width
1	29.7	185.8	63.4
2	68.6	2150.1	1871.9

Peak analysis by volume			
Peak	Area	Mean	Width
1	29.7	190.1	76.6
2	68.6	2178.8	1982.1

Peak analysis by number			
Peak	Area	Mean	Width
1	29.7	190.1	76.6
2	68.6	2178.8	1982.1
